# Relationships of serum FGF23 and α-klotho with atherosclerosis in patients with type 2 diabetes mellitus

**DOI:** 10.1186/s12933-024-02205-2

**Published:** 2024-04-15

**Authors:** Jiao Bi, Mei Zheng, Ke Li, Siwei Sun, Zihang Zhang, Nana Yan, Xueping Li

**Affiliations:** 1 Xi’an Key Laboratory for Prevention and Treatment of Common Aging Diseases, Translational and Research Centre for Prevention and Therapy of Chronic Disease, Institute of Basic and Translational Medicine, Xi’an Medical University, Xi’an, 710021 PR China; 2 The First Affiliated Hospital of Xi’an Medical College, Xi’an Medical University, Xi’an, 710021 China; 3 Shaanxi Provincial People’s Hospital, Xi’an, Shaanxi 710068 China

**Keywords:** FGF23, Klotho, T2DM, Atherosclerosis, Carotid intima-media thickness

## Abstract

**Background:**

Compelling evidence suggests that calcium/phosphorus homeostasis-related parameters may be linked to diabetes mellitus and cardiovascular events. However, few studies have investigated the association of fibroblast growth factor 23 (FGF23), α-klotho and FGF23/α-klotho ratio with atherosclerosis in patients with type 2 diabetes mellitus (T2DM).

**Objective:**

This study was designed to evaluate whether FGF23, α-klotho and FGF23/α-klotho ratio are associated with T2DM and further to explore the relationships between these three factors and atherosclerosis in Chinese patients with T2DM.

**Methods:**

Serum FGF23 and α-klotho levels were measured via an enzyme-linked immunosorbent assay (ELISA) kit, and the carotid intima-media thickness (CIMT) was assessed via high-resolution color Doppler ultrasonography. The associations of serum FGF23, α-klotho and FGF23/α-klotho ratio with atherosclerosis in T2DM patients were evaluated using multivariable logistic regression models.

**Results:**

This cross-sectional study involved 403 subjects (207 with T2DM and 196 without T2DM), 41.7% of the patients had atherosclerosis, and 67.2% of the carotid intima were thickened to a thickness greater than 0.9 mm. Compared with those in the lowest tertile, higher tertiles of FGF23 levels and FGF23/α-klotho ratio were positively associated with T2DM after adjusting for covariates, and serum α-klotho concentration was inversely correlated with T2DM (all P values < 0.01). Moreover, elevated serum FGF23 levels and FGF23/α-klotho ratio were positively associated with CIMT and carotid atherosclerosis in T2DM patients (all P values < 0.01). Further spline analysis similarly revealed linear dose‒response relationship (all P values < 0.01). And there was still significant differences in CIMT and carotid atherosclerosis between the highest group of α-klotho and the reference group in T2DM patients (P values = 0.05).

**Conclusions:**

T2DM was positively linearly related to serum FGF23 concentration and FGF23/α-klotho ratio, and negatively correlated with serum α-klotho concentration. Furthermore, both FGF23 and FGF23/α-klotho ratio were positively correlated with CIMT and atherosclerosis in T2DM patients, while α-klotho was inversely correlated with both CIMT and atherosclerosis, although the associations were not completely significant. Prospective exploration and potential mechanisms underlying these associations remain to be further elucidated.

**Supplementary Information:**

The online version contains supplementary material available at 10.1186/s12933-024-02205-2.

## Introduction

With the rapid urbanization and modernization in China, type 2 diabetes mellitus (T2DM) has become an important health issue and chronic non-communicable disease with the highest morbidity and mortality rates, imposing heavy burden on individuals and society [1]. Atherosclerosis, a condition characterized by arterial plaque accumulation, is the leading cause of diabetic macrovascular disease and accounts for more than 80% of deaths and disability among individuals with T2DM [2, 3]. T2DM-related atherosclerosis is proverbial to ultimately cause various adverse cardiovascular outcomes, and it was much more harmful than simple atherosclerosis due to its increased lipid content and plaque instability [4–6]. Therefore, it is critical to identify patients with T2DM who are at a high risk of atherosclerosis so that intensive treatment strategies can be provided.

Calcium/phosphorus homeostasis-related parameters such as fibroblast growth factor 23 (FGF23) and α-klotho have been implicated in atherosclerosis [7–10], among which FGF23 was synthesized and secreted mainly by osteocytes and was primarily known for its roles in regulating phosphate and vitamin D metabolism [11], and klotho (a co-receptor of FGF23) is a low-molecular-weight type I transmembrane protein, mainly consisting of α-klotho protein and β-klotho protein, which is expressed in the distal convoluted tubules of the nephrons and certain brain regions and co-acted with FGF23 [12, 13]. Recent studies have demonstrated that serum FGF23 and α-klotho levels were associated with vascular calcification and endothelial dysfunction, both of which were key factors involved in atherosclerosis [14–18], and elevated FGF23 and decreased α-klotho levels were found to be independently associated with unstable plaques, carotid intima-media thickness (CIMT) and epicardial fat thickness (EFT) in the general populations [19–24], yet few focused on these relationships in T2DM patients, and still researchers argued that such relationships actually did not exist [25–28]. FGF23 appeared to be toxic to endothelial cells, whereas α-klotho appeared to be protective, and the inhibitory effect of FGF23 on α-klotho prompted us to further investigate the effect of the FGF23/α-klotho ratio on atherosclerosis in T2DM, which has received little attention [29]. Therefore, due to the limited and conflicting evidence in the domestic population, it remains unclear whether FGF23, α-klotho and FGF23/α-klotho ratio are related to atherosclerosis in patients with T2DM.

Based on the above research background, the purpose of the present study was twofold. First, we evaluated whether FGF23, α-klotho and FGF23/α-klotho ratio are associated with T2DM. Second, we explored the relationships of FGF23, α-klotho and FGF23/α-klotho ratio with atherosclerosis in Chinese patients with T2DM, and whether FGF23 or α-klotho can be diagnostic and predictive biomarkers for atherosclerosis in T2DM patients.

## Materials and methods

### Study population

A total of 207 patients hospitalized and diagnosed as newly diagnosed T2DM were from the Department of Endocrinology of Shaanxi Provincial People’s Hospital from April 2022 to September 2022 were collected, and 196 healthy people in the physical examination center of the hospital were collected as control group during the same period. All participants provided written informed consent. This investigation was approved by the Medical Ethics Committee of the School of Xi’an Medical College.

**Fig. 1:**
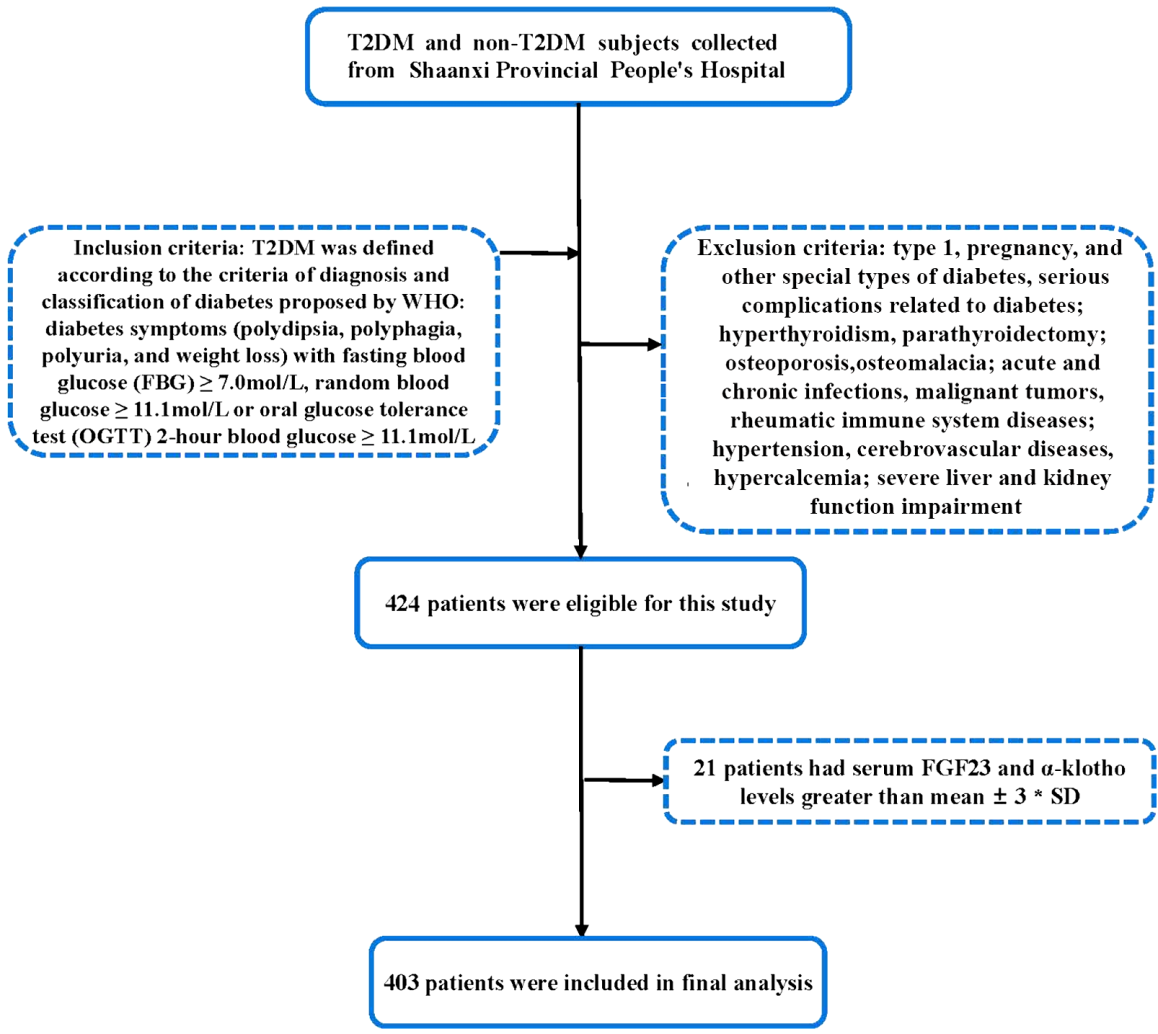
Flowchart of the study population. T2DM, type 2 diabetes mellitus; FGF23, fibroblast growth factor 23; α-klotho, α-membrane binding receptor Klotho; SD, standard deviation

The inclusion criteria for patients were as follows: T2DM was defined according to the criteria of diagnosis and classification of diabetes proposed by WHO: diabetes symptoms (polydipsia, polyphagia, polyuria, and weight loss) with fasting blood glucose (FBG) ≥ 7.0 mol/L, random blood glucose ≥ 11.1 mol/L or oral glucose tolerance test (OGTT) 2-hour blood glucose ≥ 11.1 mol/L. The exclusion criteria for patients were as follows: Subjects with type 1, pregnancy, and other special types of diabetes, serious complications related to diabetes; hyperthyroidism, parathyroidectomy; osteoporosis, osteomalacia; acute and chronic infections, malignant tumors, rheumatic immune system diseases; hypertension, cerebrovascular diseases, hypercalcemia; severe liver and kidney function impairment were excluded. Overall, 403 patients were included in this study (Fig. 1).

### Measurement of serum FGF23 and α-klotho levels

Human FGF23 ELISA Kit (Beyotime) and Human α-klotho ELISA Kit (Solarbio) were used to detect the corresponding protein concentrations respectively, which were both based on double antibody sandwich enzyme-linked immunosorbent assay (ELISA), and serum samples were sent to Wuhan Xavier Biological Laboratory for detection, and the detailed steps are as follows;

Peripheral venous blood was taken after overnight fasting, and the serum samples were separated and frozen at − 80 °C for subsequent analysis. Serum samples were firstly diluted in dilution buffer without carrier protein and then added to 96-well microplates (100 µL). After the plate was sealed, the plates were incubated at room temperature. Afterwards, the wells were washed five times with buffer (400 µL) after containment, and each well was shielded with 300 µL of diluent. Afterwards, the plates were incubated at room temperature for at least 1 h and washed several times so that the antibody coating was completed. Then, Streptaviin-ORP (100 µL), a specific sandwich of the FGF23 or α-klotho immune complex, was added and incubated for 20 min in the dark. Finally, the substrate (100 µL) and termination solution (50 µL) were added, after which the mixture was fully mixed. The absorbance (OD) of each well was determined by a microplate reader (450 nm) for FGF23 and α-klotho, and the corresponding concentrations of FGF23 and α-klotho were calculated from the standard curve. The quality control results showed a variation coefficient of less than 10% in and between plates and a recovery rate of more than 95%.

### Carotid artery-intimal thickness and carotid plaque measurement

The cervical vessels were examined by experienced physicians. All patients underwent color Doppler ultrasound examination with a Philips IU22 color Doppler ultrasound diagnostic instrument, and the linear array probe frequency was 7.0 MHz. Patients with carotid plaques were placed in the supine position, the supraclavicular fossa was used as the starting point for scanning, and the initial segment and bifurcation of the carotid artery were scanned. Once plaque was found, the local carotid artery was scanned repeatedly to evaluate the location, size, and number of carotid plaques. The above procedure was repeated to explore the condition of the bilateral carotid arteries of the patient. The diagnostic criteria for carotid atherosclerosis were formulated by the National Conference of Internal Medicine, and the results of carotid intima ultrasound examination were divided into three categories: (1) Normal blood vessels: CIMT was thin and smooth, as indicated by a double line sign, and CIMT thickness ≤ 0.9 mm; (2) CIMT thickening: Ultrasound revealed irregular CIMT echoes with thick light spots and an CIMT thickness of 0.9–1.2 mm; (3) Carotid plaque formation: Ultrasound showed that the vessel wall of the lesion site was elevated into the lumen, and the CIMT thickness at the lesion site was ≥ 1.2 mm or 1.5 times greater than that at the adjacent site.

### Definition of covariates

The basic information (name, age, BMI, blood pressure, smoking status, and duration of diabetes) of the patients was collected. BMI was calculated as weight (kg) divided by height squared (m^2^). Smoking status is defined as smoking and never smoking, while smoking includes current smoking or former smoked. Biochemical indicators, including fasting blood glucose (FBG), glycosylated hemoglobin (HbA1c), systolic blood pressure (SBP), diastolic blood pressure (DBP), triglycerides (TG), total cholesterol (TC), high-density lipoprotein cholesterol (HDL-c), low-density lipoprotein cholesterol (LDL-c), blood urea nitrogen (BUN), serum creatinine (Scr), cystatin C (Cys-C) and 25-hydroxyvitamin D3 (25-(OH)D3), were detected in blood and urine samples collected from the Department of Clinical Laboratory of Shaanxi Provincial People’s Hospital. And eGFR was calculated based on the serum cystatin C (Cys-C) concentration and creatinine concentration according to the combined creatinine–cystatin C equation, which is useful as a confirmatory test for chronic kidney disease [30].

### Statistical analysis

Since the distributions of FGF23 and α-klotho levels were both skewed, these two variables were log10-transformed to obtain normal distributions. Participants were then divided into 3 groups based on tertile cut-off values for serum FGF23 and α-klotho levels. Baseline information was expressed as the mean and standard deviation (SD) or median and interquartile ranges (IQRs) for continuous variables and the number (percentage) of categorical variables based on tertiles of serum FGF23 and α-klotho.

Using multiple logistic regression models, we estimated odds ratios (ORs) with 95% confidence intervals (CIs) for serum FGF23, α-klotho levels and FGF23/α-klotho ratio in patients with T2DM and carotid atherosclerosis and per unit increase in log10-transformed FGF23, α-klotho levels and FGF23/α-klotho ratio. Age, sex, smoking status (smoking and never smoking), BMI, SBP, DBP, TC, TG, HDL-c, LDL-c, and eGFR were used as confounders in this analysis. We excluded 21 samples with detection concentrations of FGF23 and α-klotho exceeded the median plus or minus 1.5 standard deviations, and a total of 18 subjects had missing covariate data and were estimated using multiple interpolation. In addition, we conducted spline analyses to explore the dose‒response relationships of serum FGF23, α-klotho levels and FGF23/α-klotho ratio with T2DM, T2DM combined with CIMT and T2DM combined with carotid atherosclerosis, with 3 knots placed at the 5th, 50th, and 95th percentiles and the reference value set at the 10th percentile of serum FGF23, α-klotho levels and FGF23/α-klotho ratio.

All analyses were completed with R (version 3.6.1, R Foundation for Statistical Computing). Two-sided P values less than 0.05 were considered to indicate statistical significance.

## Results

### Population characteristics

The demographic information and clinical indicators according to T2DM status were summarized in Table 1. A total of 403 participants were included in the analysis with a mean age of 54 years old and a mean FBG of 5.6 mmoL. Males made up 58.3% (n = 235) of the total population and 37.2% (n = 150) were smokers. The median and IQR of the serum FGF23 concentration in the total population were 353.3 (238.7, 484.9) pg/ml, and the median and IQR of the serum α-klotho concentration were 2.5 (1.4, 4.6) pg/ml. Among patients with T2DM, 87 individuals had carotid atherosclerosis. Compared with those who without T2DM, T2DM patients had higher TG, FBG, HbA1c, BUN, Cys-C, and FGF23 levels and lower HDL-c, 25- (OH)D3, Scr, Ca, P and α-klotho levels. However, sex, smoking status, BMI, SBP, DBP, TC, LDL-c and eGFR did not significantly differ among participants.

**Table 1:** General baseline characteristics of study participants

Characteristics	Total (n = 403)	T2DM (n = 207)	Non-T2DM (n = 196)	*P* value
Age (years)	54.0 (12.0)	53.2 (15.0)	53.5 (10.0)	0.62
Males, n (%)	235 (58.3)	126 (60.9)	109 (55.6)	0.17
Smoking status, n (%)				0.54
Never smoking	253 (62.8)	130 (62.8)	123 (62.8)	
Smoking	150 (37.2)	77 (37.3)	73 (37.2)	
BMI (kg/m²)	24.2 (4.7)	24.9 (4.7)	24.6 (4.2)	0.85
SBP (mmHg)	119.0 (14.0)	117.1 (12.3)	118.6 (14.0)	0.07
DBP (mmHg)	75.0 (8.0)	75.6 (9.0)	74.4 (7.0)	0.07
TC (mM)	5.0 (1.5)	4.8 (1.5)	5.0 (1.4)	0.08
TG (mM)	1.7 (1.2)	2.2 (1.4)	1.7 (0.8)	**< 0.01**
HDL-c (mM)	1.1 (0.4)	1.1 (0.3)	1.3 (0.4)	**< 0.01**
LDL-c (mM)	2.8 (1.0)	2.8 (1.1)	2.9 (1.0)	0.09
FBG (mM)	5.6 (4.1)	9.9 (4.1)	5.1 (0.5)	**< 0.01**
HbA1c (%)	6.0 (3.6)	8.9 (3.7)	5.4 (0.4)	**< 0.01**
25- (OH)D3 (ng/ml)	16.0 (6.4)	15.6 (7.5)	17.4 (5.0)	**0.01**
Scr (mM)	57.0 (20.2)	55.4 (19.7)	63.4 (21.9)	**< 0.01**
BUN (mM)	4.9 (1.7)	5.5 (1.8)	4.6 (1.3)	**< 0.01**
Cys-C (mM)	0.81 (0.2)	0.90 (0.2)	0.80 (0.2)	**< 0.01**
Ca (mM)	2.34 (0.11)	2.34 (0.13)	2.36 (0.12)	**0.02**
P (mM)	1.2 (0.3)	1.1 (0.2)	1.4 (0.4)	**< 0.01**
eGFR (ml/min/1.73m^2^)	101.5 (19.7)	99.6 (22.6)	101.3 (18.7)	0.40
FGF23 (pg/ml)	353.3 (246.2)	451.7 (192.6)	308.4 (195.3)	**< 0.01**
α-klotho (ng/ml)	2.5 (3.2)	2.8 (2.3)	3.6 (3.5)	**< 0.01**

*Abbreviations* T2DM, type 2 diabetes mellitus; BMI, body mass index; SBP, systolic blood pressure; DBP, diastolic blood pressure; TC, total cholesterol; TG, triglyceride; HDL-c, high-density lipoprotein cholesterol; LDL-c, low-density lipoprotein cholesterol; FBG, fasting blood glucose; HbA1c, glycosylated hemoglobin; 25-(OH)D3, 25-hydroxyvitamin D3; Scr, creatinine; BUN, blood urea nitrogen; Cys-C, cystatin C; eGFR, estimated glomerular filtration rate; FGF23, fibroblast growth factor 23; α-klotho, α-membrane binding receptor Klotho

Continuous data are presented as the mean (SD) or median (IQR) according to their distribution and as the number (percentage) for categorical variables (males and smoking status)

Bold font indicates statistical significance

### Associations of serum FGF23, α-klotho levels and FGF23/α-klotho ratio with T2DM

**Fig. 2:**
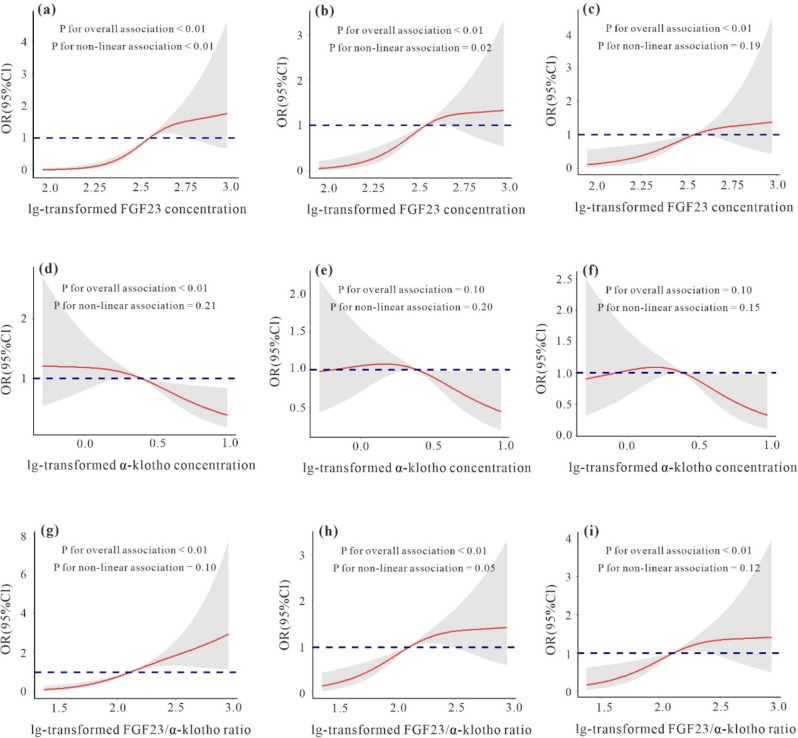
The restricted cubic splines for the associations between levels of serum FGF23 (lg-transformed), α-klotho (lg-transformed) levels and FGF23/α-klotho ratio (lg-transformed) with T2DM (**a**, **d**, **g**), T2DM combined with CIMT (**b**, **e**, **h**) and T2DM combined with atherosclerosis (**c**, **f**, **i**). The solid lines represent the adjusted change in each indicator, the dashed lines are the 95% confidence intervals (CIs) based on restricted cubic splines for serum FGF23 (lg-transformed), α-klotho (lg-transformed) levels and FGF23/α-klotho ratio (lg-transformed) with knots at the 5th, 50th, and 95th percentiles, and the reference values were set at the 10th percentiles. The models were adjusted for age, sex, BMI, smoking status, SBP, DBP, TC, TG, HDL-c, LDL-c and eGFR

**Fig. 3:**
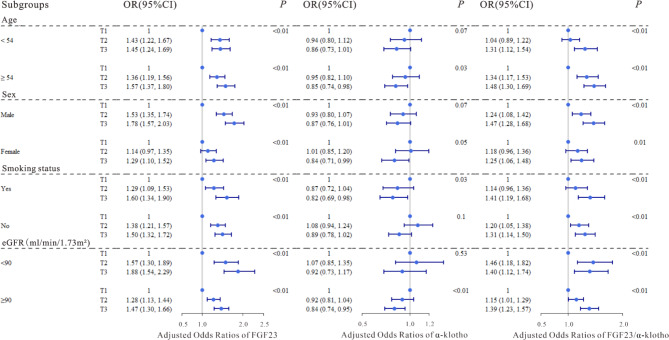
Adjusted odds ratios (95% confidence intervals [CIs]) for T2DM according to tertiles of serum FGF23, α-klotho levels, and FGF23/α-klotho ratio in subgroups stratified by age, sex, smoking status, and eGFR. The blue dots represented OR values, and the dark blue lines represented 95% confidence intervals. The models were adjusted for age, sex, BMI, smoking status, SBP, DBP, TC, TG, HDL-c, LDL-c and eGFR

We applied logistic regression models to evaluate the associations of the serum FGF23, α-klotho levels and FGF23/α-klotho ratio with T2DM, and the results were shown in Table 2. After multivariate adjustment, the 2nd (OR = 1.36, 95% CI: 1.23, 1.51; OR = 1.22, 95% CI: 1.10, 1.36) and the 3rd tertiles (OR = 1.59, 95% CI: 1.43, 1.77; OR = 1.38, 95% CI: 1.24, 1.54) were significantly positively correlated with T2DM compared with the lowest tertile of serum FGF23 and FGF23/α-klotho ratio (all P-trend < 0.05), and significant negative correlations were observed between serum α-klotho and T2DM in the both 2nd (OR = 0.97, 95% CI: 0.88, 1.08) and 3rd tertiles (OR = 0.85, 95% CI: 0.77, 0.94) compared with the reference group (all P-trend < 0.05), similar results were also observed when serum FGF23, α-klotho levels and FGF23/α-klotho ratio were dichotomized (Table S1). The restricted cubic splines showed that the serum FGF23, α-klotho levels and FGF23/α-klotho ratio were linearly correlated with T2DM (P for overall < 0.05; Fig. 2a, d, g). After stratification, we explored differences in various subgroups according to age (< 54 y, ≥ 54 y), sex (male, female), smoking status (yes, no), and eGFR (< 90 ml/min/1.73m^2^, ≥ 90 ml/min/1.73m^2^), and significant differences were found in the results of most groups, α-klotho was not significantly associated with type 2 diabetes and was only found in the following populations; ≥ 54 y, male, non-smoker, with an eGFR index less than 90 ml/min/1.73m^2^ (Table S2, Fig. 3).

**Table 2:** Odds ratios (95% confidence intervals [CIs]) of T2DM according to tertiles

	Tertiles				1-unit increment of log variables	*P* value ^b^
	T1	T2	T3	*P*-trend ^a^		
**FGF23**						
Model 1	1.0 (ref)	**1.42 (1.28, 1.58)**	**1.69 (1.52, 1.89)**	**< 0.01**	**2.52 (2.10, 3.02)**	**< 0.01**
Model 2	1.0 (ref)	**1.42 (1.28, 1.59)**	**1.71 (1.53, 1.90)**	**< 0.01**	**2.57 (2.14, 3.08)**	**< 0.01**
Model 3	1.0 (ref)	**1.36 (1.23, 1.51)**	**1.59 (1.43, 1.76)**	**< 0.01**	**2.23 (1.87, 2.66)**	**< 0.01**
Model 4	1.0 (ref)	**1.36 (1.23, 1.51)**	**1.59 (1.43, 1.77)**	**< 0.01**	**2.23 (1.87, 2.67)**	**< 0.01**
**α-klotho**						
Model 1	1.0 (ref)	0.97 (0.86, 1.08)	**0.83 (0.75, 0.93)**	**< 0.01**	**0.80 (0.71, 0.91)**	**< 0.01**
Model 2	1.0 (ref)	0.97 (0.87, 1.08)	**0.84 (0.75, 0.93)**	**< 0.01**	**0.81 (0.71, 0.92)**	**< 0.01**
Model 3	1.0 (ref)	0.97 (0.88, 1.08)	**0.85 (0.77, 0.94)**	**< 0.01**	**0.82 (0.72, 0.92)**	**< 0.01**
Model 4	1.0 (ref)	0.97 (0.88, 1.08)	**0.85 (0.77, 0.94)**	**< 0.01**	**0.82 (0.72, 0.92)**	**< 0.01**
**FGF23/α-klotho**						
Model 1	1.0 (ref)	**1.26 (1.12, 1.41)**	**1.45 (1.29, 1.63)**	**< 0.01**	**1.56 (1.40, 1.74)**	**< 0.01**
Model 2	1.0 (ref)	**1.26 (1.12, 1.41)**	**1.45 (1.29, 1.63)**	**< 0.01**	**1.56 (1.40, 1.75)**	**< 0.01**
Model 3	1.0 (ref)	**1.22 (1.10, 1.36)**	**1.38 (1.24, 1.54)**	**< 0.01**	**1.47 (1.33, 1.64)**	**< 0.01**
Model 4	1.0 (ref)	**1.22 (1.10, 1.36)**	**1.38 (1.24, 1.54)**	**< 0.01**	**1.47 (1.33, 1.64)**	**< 0.01**

Bold font indicates statistical significance

Model 1: adjusted for age and sex

Model 2: based on Model 1, additionally adjusted for smoking status and BMI.

Model 3: based on Model 2, additionally adjusted for SBP, DBP, TC, TG, HDL-c, and LDL-c.

Model 4: based on Model 3, additionally adjusted for eGFR.

*Abbreviations* T2DM, type 2 diabetes mellitus; FGF23, fibroblast growth factor 23; α-klotho, α-membrane binding receptor Klotho

^a^*P*-trend was obtained by including the median of each quartile (log_10_-transformed) of serum FGF23, α-klotho or FGF23/α-klotho ratio as a continuous variable in the logistic regression models

^b^*P* value was linear for each 1-unit increase in log_10_ FGF23, α-klotho or FGF23/α-klotho ratio

### Associations of serum FGF23, α-klotho levels and FGF23/α-klotho ratio with T2DM combined with atherosclerosis

**Fig. 4:**
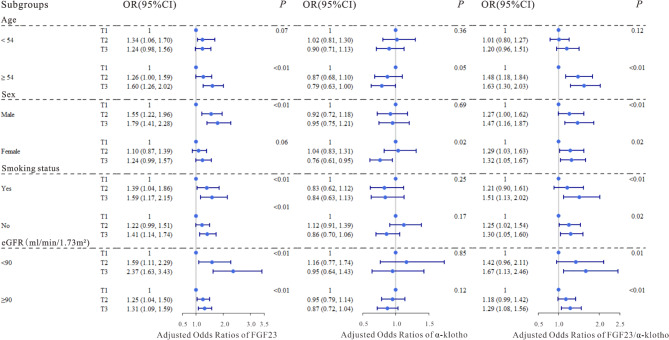
Adjusted odds ratios (95% confidence intervals [CIs]) for T2DM combined with CIMT according to tertiles of serum FGF23, α-klotho levels, and FGF23/α-klotho ratio in subgroups stratified by age, sex, smoking status, and eGFR. The blue dots represented OR values, and the dark blue lines represented 95% confidence intervals. The models were adjusted for age, sex, BMI, smoking status, SBP, DBP, TC, TG, HDL-c, LDL-c and eGFR

**Fig. 5:**
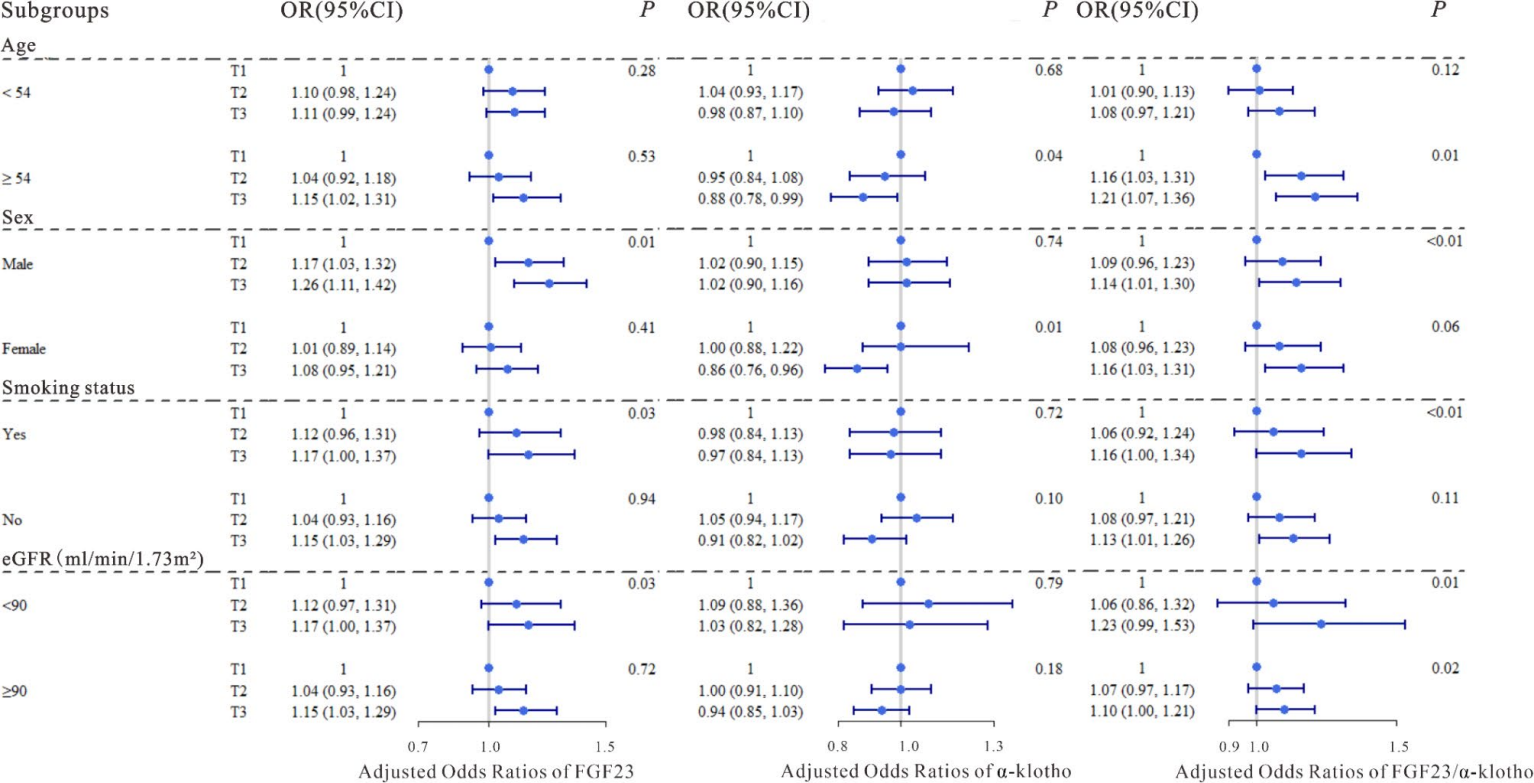
Adjusted odds ratios (95% confidence intervals [CIs]) for T2DM combined with atherosclerosis according to tertiles of serum FGF23, α-klotho levels, and FGF23/α-klotho ratio in subgroups stratified by age, sex, smoking status, and eGFR. The blue dots represented OR values, and the dark blue lines represented 95% confidence intervals. The models were adjusted for age, sex, BMI, smoking status, SBP, DBP, TC, TG, HDL-c, LDL-c and eGFR

To investigate whether serum FGF23, α-klotho levels and FGF23/α-klotho ratio were associated with T2DM combined with atherosclerosis, we fitted ordered regression models and logistic regression models to assess the associations of the factors with atherosclerosis in patients with T2DM (Table 3). Compared with those in the lowest quartile of serum FGF23 and FGF23/α-klotho ratio, significant positive associations were observed between the serum FGF23, FGF23/α-klotho ratio and CIMT in both 2nd (OR = 1.32, 95% CI: 1.12, 1.56; OR = 1.56, 95% CI: 1.31, 1.84) and 3rd tertiles (OR = 1.26, 95% CI: 1.07, 1.49; OR = 1.38, 95% CI: 1.17, 1.63) after multivariable adjustment (all P-trend < 0.05), and a significant negative association was observed between the serum α-klotho concentration and CIMT in the 3rd tertile (OR = 0.85, 95% CI: 0.72, 1.00) in Model 3. When serum FGF23, α-klotho levels and FGF23/α-klotho ratio were divided into two categories, the results were statistically significant (Table 3). The restricted cubic splines revealed significant linear associations of serum FGF23 levels and FGF23/α-klotho ratio with T2DM combined with CIMT (P for overall < 0.01; Fig. 2b and h), but no significant association was found between serum α-klotho levels and T2DM combined with CIMT (Fig. 2e). Furthermore, we divided the dependent variables into those with carotid atherosclerosis and those without atherosclerosis, and the results were similar to those described above, suggesting that our results were stable (Table 4, S4), and the restricted cubic splines indicated significant linear associations of both serum FGF23 levels and FGF23/α-klotho ratio with atherosclerosis (P for overall < 0.05; Fig. 2c and i), and no significant association was also found between serum α-klotho levels and T2DM combined with atherosclerosis (Fig. 2f). In stratified analyses, FGF23 and FGF23/α-klotho ratio were both significantly related to T2DM combined with CIMT in the lowest- and highest-concentration groups of all strata, except for those < 54 y, and significant negative association between α-klotho and T2DM combined with CIMT was found only in males and those who ≥ 54 y (Table S5, Fig. 4), moreover, similar stratified results were observed among serum FGF23, α-klotho levels and FGF23/α-klotho ratio with T2DM combined with atherosclerosis (Table S6, Fig. 5).

**Table 3:** Odds ratios (95% confidence intervals [CIs]) of T2DM patients combined with CIMT according to tertiles

	Tertiles				1-unit increment of log variables	*P* value ^b^
	T1	T2	T3	*P*-trend ^a^		
**FGF23**						
Model 1	1.0 (ref)	**1.50 (1.25, 1.80)**	**1.75 (1.46, 2.11)**	**< 0.01**	**2.72 (2.00, 3.69)**	**< 0.01**
Model 2	1.0 (ref)	**1.49 (1.24, 1.79)**	**1.76 (1.47, 2.12)**	**< 0.01**	**2.77 (2.03, 3.77)**	**< 0.01**
Model 3	1.0 (ref)	**1.32 (1.12, 1.56)**	**1.56 (1.32, 1.84)**	**< 0.01**	**2.16 (1.62, 2.88)**	**< 0.01**
Model 4	1.0 (ref)	**1.32 (1.12, 1.56)**	**1.56 (1.31, 1.84)**	**< 0.01**	**2.16 (1.62, 2.87)**	**< 0.01**
**α-klotho**						
Model 1	1.0 (ref)	1.00 (0.83, 1.20)	**0.84 (0.70, 1.00)**	0.07	0.83 (0.67, 1.03)	0.09
Model 2	1.0 (ref)	1.01 (0.84, 1.21)	0.84 (0.70, 1.01)	0.07	0.84 (0.67, 1.04)	0.10
Model 3	1.0 (ref)	0.99 (0.84, 1.17)	**0.85 (0.72, 1.00)**	0.06	0.83 (0.68, 1.01)	0.06
Model 4	1.0 (ref)	0.99 (0.84, 1.17)	0.85 (0.73, 1.01)	0.06	0.83 (0.69, 1.01)	0.06
**FGF23/α-klotho**						
Model 1	1.0 (ref)	**1.34 (1.12, 1.62)**	**1.50 (1.25, 1.81)**	**< 0.01**	**1.56 (1.30, 1.88)**	**< 0.01**
Model 2	1.0 (ref)	**1.34 (1.11, 1.61)**	**1.51 (1.25, 1.81)**	**< 0.01**	**1.57 (1.30, 1.88)**	**< 0.01**
Model 3	1.0 (ref)	**1.26 (1.07, 1.49)**	**1.38 (1.17, 1.63)**	**< 0.01**	**1.44 (1.22, 1.70)**	**< 0.01**
Model 4	1.0 (ref)	**1.26 (1.07, 1.49)**	**1.38 (1.17, 1.63)**	**< 0.01**	**1.44 (1.22, 1.70)**	**< 0.01**

Bold font indicates statistical significance

Model 1: adjusted for age and sex

Model 2: based on Model 1, additionally adjusted for smoking status and BMI.

Model 3: based on Model 2, additionally adjusted for SBP, DBP, TC, TG, HDL-c, and LDL-c.

Model 4: based on Model 3, additionally adjusted for eGFR.

*Abbreviations* T2DM, type 2 diabetes mellitus; FGF23, fibroblast growth factor 23; α-klotho, α-membrane binding receptor Klotho

^a^*P*-trend was obtained by including the median of each quartile (log_10_-transformed) of serum FGF23, α-klotho or FGF23/α-klotho ratio as a continuous variable in the logistic regression models

^b^*P* value was linear for each 1-unit increase in log_10_ FGF23, α-klotho or FGF23/α-klotho ratio

**Table 4:** Odds ratios (95% confidence intervals [CIs]) of T2DM combined with atherosclerosis according to tertiles

		Tertiles			1-unit increment of log variables	*P* value ^b^
	T1	T2	T3	*P*-trend ^a^		
**FGF23**						
Model 1	1.0 (ref)	**1.16 (1.06, 1.27)**	**1.25 (1.14, 1.38)**	**< 0.01**	**1.48 (1.26, 1.73)**	**< 0.01**
Model 2	1.0 (ref)	**1.16 (1.06, 1.27)**	**1.26 (1.15, 1.38)**	**< 0.01**	**1.50 (1.28, 1.76)**	**< 0.01**
Model 3	1.0 (ref)	**1.10 (1.00, 1.19)**	**1.19 (1.09, 1.30)**	**< 0.01**	**1.34 (1.15, 1.55)**	**< 0.01**
Model 4	1.0 (ref)	**1.10 (1.00, 1.20)**	**1.19 (1.09, 1.30)**	**< 0.01**	**1.34 (1.15, 1.55)**	**< 0.01**
**α-klotho**						
Model 1	1.0 (ref)	1.03 (0.94, 1.13)	0.93 (0.85, 1.02)	0.14	0.92 (0.82, 1.02)	0.13
Model 2	1.0 (ref)	1.03 (0.94, 1.13)	0.93 (0.85, 1.02)	0.15	0.92 (0.82, 1.03)	0.14
Model 3	1.0 (ref)	1.02 (0.93, 1.11)	0.94 (0.86, 1.01)	0.12	0.91 (0.83, 1.01)	0.08
Model 4	1.0 (ref)	1.02 (0.94, 1.11)	0.94 (0.86, 1.02)	0.13	0.91 (0.83, 1.01)	0.09
**FGF23/α-klotho**						
Model 1	1.0 (ref)	**1.12 (1.02, 1.22)**	**1.18 (1.07, 1.29)**	**< 0.01**	**1.20 (1.10, 1.32)**	**< 0.01**
Model 2	1.0 (ref)	**1.11 (1.01, 1.22)**	**1.18 (1.07, 1.29)**	**< 0.01**	**1.21 (1.10, 1.32)**	**< 0.01**
Model 3	1.0 (ref)	1.08 (0.99, 1.18)	**1.13 (1.04, 1.23)**	**< 0.01**	**1.16 (1.07, 1.27)**	**< 0.01**
Model 4	1.0 (ref)	1.08 (0.99, 1.18)	**1.13 (1.01, 1.23)**	**< 0.01**	**1.16 (1.07, 1.27)**	**< 0.01**

Bold font indicates statistical significance

Model 1: adjusted for age and sex

Model 2: based on Model 1, additionally adjusted for smoking status and BMI.

Model 3: based on Model 2, additionally adjusted for SBP, DBP, TC, TG, HDL-c, and LDL-c.

Model 4: based on Model 3, additionally adjusted for eGFR

*Abbreviations* T2DM, type 2 diabetes mellitus; FGF23, fibroblast growth factor 23; α-klotho, α-membrane binding receptor Klotho

^a^*P*-trend was obtained by including the median of each quartile (log_10_-transformed) of serum FGF23, α-klotho or FGF23/α-klotho ratio as a continuous variable in the logistic regression models

^b^*P* value was linear for each 1-unit increase in log_10_ FGF23, α-klotho or FGF23/α-klotho ratio

### ROC curve prediction analysis of serum FGF23, α-klotho levels and FGF23/α-klotho ratio with T2DM and T2DM combined with atherosclerosis

**Fig. 6:**
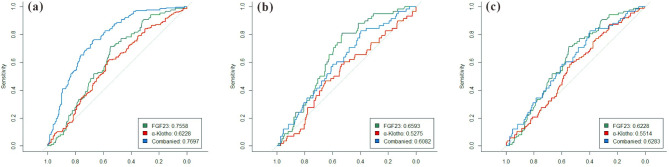
ROC curve prediction analysis of the serum FGF23 and α-klotho levels, and their combination in patients with T2DM (**a**), patients with T2DM combined with CIMT (**b**) and patients with T2DM combined with atherosclerosis (**c**). The green curves represented the prediction probability of FGF23, the red curves represented the prediction probability of α-klotho, and the blue curves represented the prediction probability of joint detection rent. The larger the area under the curve, the stronger the prediction probability

Based on the results of the ROC curve analysis (Fig. 6), we found that the predictive effect of the combined group of FGF23 and α-klotho (area under the curve [AUC]: 0.7697) was slightly higher than that of FGF23 (AUC: 0.7558) for T2DM, and the AUC of α-klotho was the lowest (AUC: 0.5852). When predicting T2DM combined with CIMT, the AUC of FGF23 was the highest (AUC: 0.6593), likewise, the combined group maintained the highest prediction probability (AUC: 0.6283) when we predicted T2DM combined with atherosclerosis. The results suggested that the combination of FGF23 and α-klotho not only improved the prediction probability of T2DM, but also improved the diagnosis rate of T2DM combined with atherosclerosis.

## Discussion

The present study indicated positive associations between serum FGF23 levels and FGF23/α-klotho ratio with T2DM and a negative relationship between α-klotho with T2DM, and the results showed that serum FGF23 and FGF23/α-klotho ratio were both positively associated with atherosclerosis in individuals with T2DM while α-klotho was inversely correlated, postulating that FGF23 elevations and α-klotho reductions may influence the development or progression of atherosclerosis in T2DM patients.

To date, limited human evidence has been found to directly support a connection between FGF23 and α-klotho with T2DM. However, only a few clinical studies have shown associations between FGF23, α-klotho and various indexes related to T2DM, including insulin resistance (measured by homeostasis model assessment of insulin resistance), dyslipidemia, resistin levels, and obesity status, suggesting that an imbalance in the FGF23/α-klotho axis may result in an unfavorable lipid profile, which could contribute to the development or progression of both T2DM and atherosclerosis [31–33]. Although several studies have shown a negative correlation between α-klotho and diabetes [34, 35], recent published studies have ruled out this association [28, 36]. The present study demonstrated positive correlations between serum FGF23 levels and FGF23/α-klotho ratio with T2DM and a negative correlation between serum α-klotho levels and T2DM. We used 3 models to enhances the stability, reliability, and robustness of analysis, and we specifically adjusted for estimated eGFR in our fourth analytical model in order to make the model more comprehensive, and the results were not obviously altered [37]. Mechanistic studies revealed that the FGF23/α-klotho axis could directly induce diabetes by affecting the intracellular pathway or α-carboxylase axis to regulate the fat content and distribution in hepatocytes and skeletal muscle cells [38] and indirectly alter glucose tolerance through the induction of calcium and phosphorus metabolism disorders through phosphorus modulation [39].

Previous clinical studies have shown that FGF23 and α-klotho have highly valuable predictive effects on cardiovascular disease (CVD) in patients with T2DM [40–45], but atherosclerosis, the initial cause of cardiovascular events, has received relatively little attention [46]. To date, only three observational analyses of T2DM patients revealed that FGF23 was significantly associated with unstable plaques, vascular calcification and CIMT. A retrospective analysis firstly revealed that FGF23 was significantly and independently associated with unstable plaques in patients with T2DM who underwent carotid endarterectomy [19], and similar results were also found in African-American T2DM patients without end-stage renal disease [20]. In addition, researchers from Turkey believed that FGF23 can also be used as a noninvasive predictor of subclinical atherosclerosis in patients with gestational diabetes [47]. Moreover, low α-klotho levels were associated with increased CIMT and EFT, yet these studies were not conducted in people with diabetes [22, 23]. Interestingly, high α-klotho levels have been observed to be positively associated with carotid atherosclerosis in diabetic patients, which was different from the above results [28]. In our current study, we observed that serum FGF23 levels and FGF23/α-klotho ratio were positively correlated with carotid atherosclerosis in T2DM patients, while serum α-klotho levels were negatively associated with carotid atherosclerosis in T2DM patients, suggesting that FGF23 might be an independent risk factor for adverse cardiovascular events and that α-klotho might be a protective factor, likewise, FGF23/α-klotho ratio may also be a novel indicator for predicting T2DM combined with atherosclerosis [48]. Indeed, a prolonged hyperglycemic state activates oxidative stress pathways, subsequently causing vascular endothelial damage and ultimately leading to the formation of plaques, suggesting that the relationship between FGF23 or α-klotho and atherosclerosis may be partially driven by hyperglycemia [49]. More specifically, the association may be partially explained by the involvement of the FGF23/α-klotho axis in the complex process of vascular calcification via the Wnt7b/β-catenin signaling pathway [15]. For instance, FGF23 appears to alter levels of calcium and phosphate metabolism through the (extracellular signal-regulated kinase/mitogen-activated kinase) ERK/MAPK signaling pathway, directly promoting calcification of smooth muscle cells in arterial walls or indirectly affecting endothelial function [50, 51], and the antioxidant stress function of α-klotho is a key factor in combating the progression of atherosclerosis [52, 53], and α-klotho can reduce cell apoptosis and senescence through FGFR1 and ERK1/2 pathways, change phosphate-induced vascular calcification, and ultimately affect the occurrence and development of atherosclerosis [54, 55]. With a more detailed understanding of the mechanisms underlying atherosclerosis in T2DM patients, researchers and clinicians can develop more targeted strategies for the prevention and treatment of CVD-related complications in T2DM patients.

The main advantages of this study are based on the relatively complete population information, and the ratio of FGF23 to α-klotho is also contained. The presence of FGF23 and α-klotho in patients with T2DM combined with atherosclerosis has important implications for the early prevention of T2DM and related complications. Nonetheless, there are still several limitations in the present study. First, the relatively small sample size of the study population consisting of middle-aged or elderly people limits the generalizability of the results to other populations. Second, our cross-sectional study limited us to exploring the causal association of FGF23 and α-klotho with T2DM or atherosclerosis in T2DM patients; therefore, prospective studies are needed to validate the practical relevance of these findings. Finally, residual confounding factors, such as detailed diet, daily routine and missing drug intake data, which we did not take into account, may confound our findings. Thus, epidemiological studies with larger sample size and further mechanistic studies are needed to confirm our findings in other populations. While studies have focused on mineral metabolism components such as FGF23 and α-klotho, additional prospective research is needed to fully understand the mechanisms through which FGF23 and α-klotho contribute to atherosclerosis in T2DM patients and to validate the role of FGF23/α-klotho ratio as a novel marker. These findings may have important clinical implications for the management and treatment of CVD in the future.

## Conclusions

To our knowledge, this study systemically investigated the associations of FGF23, α-klotho and FGF23/α-klotho ratio with T2DM and its associated atherosclerosis in middle-aged and elderly populations. Our results showed that the serum FGF23 concentration and FGF23/α-klotho ratio were positively associated with T2DM and T2DM combined with atherosclerosis and α-klotho inversely correlated. FGF23, α-klotho and their combination have certain predictive effects on T2DM and T2DM with atherosclerosis.

### Electronic supplementary material

Below is the link to the electronic supplementary material.

Supplementary Material 1

## Data Availability

The datasets used and/or analyzed during the current study are available from the corresponding author on reasonable request.
