# Supplementary material for: Relationships of serum FGF23 and α-klotho with atherosclerosis in patients with type 2 diabetes mellitus
Source: Cardiovasc Diabetol. 2024 Apr 15;23:128. doi: 10.1186/s12933-024-02205-2 (PMC11020347; doi:10.1186/s12933-024-02205-2)
Supplement: Supplementary file 1 — Supplementary Material 1 [file 12933_2024_2205_MOESM1_ESM.docx]

**Supplemental materials**

**Table S1.** Odds ratios (95% confidence intervals [CIs]) of T2DM according to dichotomy of serum FGF23, α-klotho levels, and FGF23/α-klotho ratio.

**Table S2.** Stratified analysis of odds ratios (95% confidence intervals [CIs]) of T2DM according to tertiles of serum FGF23, α-klotho levels, and FGF23/α-klotho ratio.

**Table S3.** Odds ratios (95% confidence intervals [CIs]) of T2DM combined with CIMT according to dichotomy of serum FGF23, α-klotho levels, and FGF23/α-klotho ratio.

**Table S4.** Odds ratios (95% confidence intervals [CIs]) of T2DM combined with carotid atherosclerosis according to dichotomy of serum FGF23, α-klotho levels, and FGF23/α-klotho ratio.

**Table S5.** Stratified analysis of odds ratios (95% confidence intervals [CIs]) of T2DM combined with CIMT according to tertiles of serum FGF23, α-klotho levels, and FGF23/α-klotho ratio.

**Table S6.** Stratified analysis of odds ratios (95% confidence intervals [CIs]) of T2DM combined with atherosclerosis according to tertiles of serum FGF23, α-klotho levels, and FGF23/α-klotho ratio.

**Table S1**

|  | Subgroups | | |
| --- | --- | --- | --- |
|  | Lower | Higher | *P*-value |
| FGF23 |  |  |  |
| Model 1 | 1.0 (ref) | **1.55 (1.42, 1.69)** | **<0.01** |
| Model 2 | 1.0 (ref) | **1.56 (1.43, 1.70)** | **<0.01** |
| Model 3 | 1.0 (ref) | **1.47 (1.35, 1.60)** | **<0.01** |
| Model 4 | 1.0 (ref) | **1.47 (1.35, 1.60)** | **<0.01** |
| α-klotho |  |  |  |
| Model 1 | 1.0 (ref) | **0.85 (0.78, 0.92)** | **<0.01** |
| Model 2 | 1.0 (ref) | **0.85 (0.78, 0.93)** | **<0.01** |
| Model 3 | 1.0 (ref) | **0.87 (0.80, 0.95)** | **<0.01** |
| Model 4 | 1.0 (ref) | **0.87 (0.80, 0.95)** | **<0.01** |
| FGF23/α-klotho |  |  |  |
| Model 1 | 1.0 (ref) | **1.32 (1.20, 1.45)** | **<0.01** |
| Model 2 | 1.0 (ref) | **1.32 (1.20, 1.45)** | **<0.01** |
| Model 3 | 1.0 (ref) | **1.26 (1.16, 1.38)** | **<0.01** |
| Model 4 | 1.0 (ref) | **1.26 (1.16, 1.38)** | **<0.01** |

Odds ratios (95% confidence intervals [CIs]) of T2DM according to dichotomy of serum FGF23, α-klotho levels, and FGF23/α-klotho ratio.

Abbreviation: T2DM, type 2 diabetic mellitus; FGF23, fibroblast growth factor 23; α-klotho, α-membrane binding receptor Klotho.

Model 1: adjusted for age and sex.

Model 2: based on model 1, additionally adjusted for smoking status, and BMI.

Model 3: based on model 2, additionally adjusted for SBP, DBP, TC, TG, HDL-c, LDL-c.

Model 4: based on model 3, additionally adjusted for eGFR.

Bold font indicates statistically significant.

**Table S2**

|  | Tertiles | | | |
| --- | --- | --- | --- | --- |
|  | T1 | T2 | T3 | *P*-trend ^a^ |
| FGF23 |  |  |  |  |
| Age |  |  |  |  |
| < 54 | 1.0 (ref) | **1.43 (1.22, 1.67)** | **1.45 (1.24, 1.69)** | **<0.01** |
| ≥54 | 1.0 (ref) | **1.36 (1.19, 1.56)** | **1.57 (1.37, 1.80)** | **<0.01** |
| Sex |  |  |  |  |
| Male | 1.0 (ref) | **1.53 (1.35, 1.74)** | **1.78 (1.57, 2.03)** | **<0.01** |
| Female | 1.0 (ref) | 1.14 (0.97, 1.35) | **1.29 (1.10, 1.52)** | **<0.01** |
| Smoking status |  |  |  |  |
| Yes | 1.0 (ref) | **1.30 (1.10, 1.54)** | **1.60 (1.34, 1.91)** | **<0.01** |
| No | 1.0 (ref) | **1.38 (1.21, 1.57)** | **1.50 (1.32, 1.72)** | **<0.01** |
| eGFR |  |  |  |  |
| < 90 | 1.0 (ref) | **1.57 (1.30, 1.89)** | **1.88 (1.54, 2.29)** | **<0.01** |
| ≥90 | 1.0 (ref) | **1.28 (1.13, 1.44)** | **1.47 (1.30, 1.66)** | **<0.01** |
| α-klotho |  |  |  |  |
| Age |  |  |  |  |
| < 54 | 1.0 (ref) | 0.94 (0.80, 1.12) | 0.86 (0.73, 1.01) | 0.07 |
| ≥54 | 1.0 (ref) | 0.95 (0.82, 1.10) | **0.85 (0.74, 0.98)** | **0.03** |
| Sex |  |  |  |  |
| Male | 1.0 (ref) | 0.93 (0.80, 1.07) | 0.87 (0.76, 1.01) | 0.07 |
| Female | 1.0 (ref) | 1.01 (0.85, 1.20) | **0.84 (0.71, 0.99)** | **0.05** |
| Smoking status |  |  |  |  |
| Yes | 1.0 (ref) | 0.87 (0.72, 1.04) | **0.82 (0.69, 0.98)** | **0.03** |
| No | 1.0 (ref) | 1.08 (0.94, 1.24) | 0.89 (0.78, 1.02) | 0.10 |
| eGFR |  |  |  |  |
| < 90 | 1.0 (ref) | 1.07 (0.85, 1.35) | 0.92 (0.73, 1.17) | 0.53 |
| ≥90 | 1.0 (ref) | **0.92 (0.81, 1.04)** | **0.84 (0.74, 0.95)** | **<0.01** |
| FGF23/α-klotho |  |  |  |  |
| Age |  |  |  |  |
| < 54 | 1.0 (ref) | 1.04 (0.89, 1.22) | **1.31 (1.12, 1.54)** | **<0.01** |
| ≥54 | 1.0 (ref) | **1.34 (1.17, 1.53)** | **1.48 (1.30, 1.69)** | **<0.01** |
| Sex |  |  |  |  |
| Male | 1.0 (ref) | **1.24 (1.08, 1.42)** | **1.47 (1.28, 1.68)** | **<0.01** |
| Female | 1.0 (ref) | 1.18 (0.99, 1.39) | **1.25 (1.06, 1.48)** | **0.01** |
| Smoking status |  |  |  |  |
| Yes | 1.0 (ref) | 1.14 (0.96, 1.36) | **1.41 (1.19, 1.68)** | **<0.01** |
| No | 1.0 (ref) | **1.20 (1.05, 1.38)** | **1.31 (1.14, 1.50)** | **<0.01** |
| eGFR |  |  |  |  |
| < 90 | 1.0 (ref) | **1.46 (1.18, 1.82)** | **1.40 (1.12, 1.74)** | **<0.01** |
| ≥90 | 1.0 (ref) | **1.15 (1.01, 1.29)** | **1.39 (1.23, 1.57)** | **<0.01** |

Stratified analysis of odds ratios (95% confidence intervals [CIs]) of T2DM according to tertiles of serum FGF23, α-klotho levels, and FGF23/α-klotho ratio.

Abbreviation: T2DM, type 2 diabetic mellitus; FGF23, fibroblast growth factor 23; α-klotho, α-membrane binding receptor Klotho. Models were adjusted for age, sex, smoking status, and BMI, SBP, DBP, TC, TG, HDL-c, LDL-c, and eGFR.

^a^ *P*-trend was obtained by including the median of each tertile (log_10_-transformed) of serum FGF23, α-klotho and FGF23/α-klotho ratio as a continuous variable in the logistic regression models.

Bold font indicates statistically significant.

**Table S3**

|  | Subgroups | | |
| --- | --- | --- | --- |
|  | Lower | Higher | *P*-value |
| FGF23 |  |  |  |
| Model 1 | 1.0 (ref) | **1.61 (1.39, 1.87)** | **<0.01** |
| Model 2 | 1.0 (ref) | **1.61 (1.39, 1.87)** | **<0.01** |
| Model 3 | 1.0 (ref) | **1.45 (1.26, 1.66)** | **<0.01** |
| Model 4 | 1.0 (ref) | **1.45 (1.26, 1.66)** | **<0.01** |
| α-klotho |  |  |  |
| Model 1 | 1.0 (ref) | **0.82 (0.71, 0.95)** | **<0.01** |
| Model 2 | 1.0 (ref) | **0.82 (0.71, 0.95)** | **<0.01** |
| Model 3 | 1.0 (ref) | **0.84 (0.74, 0.96)** | **0.01** |
| Model 4 | 1.0 (ref) | **0.84 (0.74, 0.96)** | **0.01** |
| FGF23/α-klotho |  |  |  |
| Model 1 | 1.0 (ref) | **1.33 (1.14, 1.55)** | **<0.01** |
| Model 2 | 1.0 (ref) | **1.34 (1.15, 1.56)** | **<0.01** |
| Model 3 | 1.0 (ref) | **1.24 (1.08, 1.42)** | **<0.01** |
| Model 4 | 1.0 (ref) | **1.24 (1.08, 1.42)** | **<0.01** |

Odds ratios (95% confidence intervals [CIs]) of T2DM combined with CIMT according to dichotomy of serum FGF23, α-klotho levels, and FGF23/α-klotho ratio.

Abbreviation: T2DM, type 2 diabetic mellitus; FGF23, fibroblast growth factor 23; α-klotho, α-membrane binding receptor Klotho.

Model 1: adjusted for age and sex.

Model 2: based on model 1, additionally adjusted for smoking status, and BMI.

Model 3: based on model 2, additionally adjusted for SBP, DBP, TC, TG, HDL-c, LDL-c.

Model 4: based on model 3, additionally adjusted for eGFR.

Bold font indicates statistically significant.

**Table S4**

|  | Subgroups | | |
| --- | --- | --- | --- |
|  | Lower | Higher | *P*-value |
| FGF23 |  |  |  |
| Model 1 | 1.0 (ref) | **1.21 (1.12, 1.31)** | **<0.01** |
| Model 2 | 1.0 (ref) | **1.22 (1.13, 1.31)** | **<0.01** |
| Model 3 | 1.0 (ref) | **1.15 (1.07, 1.24)** | **<0.01** |
| Model 4 | 1.0 (ref) | **1.15 (1.07, 1.24)** | **<0.01** |
| α-klotho |  |  |  |
| Model 1 | 1.0 (ref) | **0.92 (0.85, 0.99)** | **0.03** |
| Model 2 | 1.0 (ref) | **0.92 (0.86, 1.00)** | **0.04** |
| Model 3 | 1.0 (ref) | **0.93 (0.87, 1.00)** | **0.04** |
| Model 4 | 1.0 (ref) | **0.93 (0.87, 1.00)** | **0.04** |
| FGF23/α-klotho |  |  |  |
| Model 1 | 1.0 (ref) | **1.12 (1.04, 1.21)** | **<0.01** |
| Model 2 | 1.0 (ref) | **1.12 (1.04, 1.21)** | **<0.01** |
| Model 3 | 1.0 (ref) | **1.09 (1.01, 1.16)** | **0.02** |
| Model 4 | 1.0 (ref) | **1.08 (1.01, 1.16)** | **0.02** |

Odds ratios (95% confidence intervals [CIs]) of T2DM combined with atherosclerosis according to dichotomy of serum FGF23, α-klotho levels, and FGF23/α-klotho ratio.

Abbreviation: T2DM, type 2 diabetic mellitus; FGF23, fibroblast growth factor 23; α-klotho, α-membrane binding receptor Klotho.

Model 1: adjusted for age and sex.

Model 2: based on model 1, additionally adjusted for smoking status, and BMI.

Model 3: based on model 2, additionally adjusted for SBP, DBP, TC, TG, HDL-c, LDL-c.

Model 4: based on model 3, additionally adjusted for eGFR.

Bold font indicates statistically significant.

**Table S5**

|  | Tertiles | | | |
| --- | --- | --- | --- | --- |
|  | T1 | T2 | T3 | *P*-trend ^a^ |
| FGF23 |  |  |  |  |
| Age |  |  |  |  |
| < 54 | 1.0 (ref) | 1.34 (1.06, 1.70) | 1.24 (0.98, 1.56) | 0.07 |
| ≥54 | 1.0 (ref) | **1.26 (1.00, 1.59)** | **1.60 (1.26, 2.02)** | **<0.01** |
| Sex |  |  |  |  |
| Male | 1.0 (ref) | **1.55 (1.22, 1.96)** | **1.79 (1.41, 2.28)** | **<0.01** |
| Female | 1.0 (ref) | 1.10 (0.87, 1.39) | 1.24 (0.99, 1.57) | 0.06 |
| Smoking status |  |  |  |  |
| Yes | 1.0 (ref) | **1.39 (1.04, 1.86)** | **1.59 (1.17, 2.15)** | **<0.01** |
| No | 1.0 (ref) | 1.22 (0.99, 1.51) | **1.41 (1.14, 1.74)** | **<0.01** |
| eGFR |  |  |  |  |
| < 90 | 1.0 (ref) | **1.59 (1.11, 2.29)** | **2.37 (1.63, 3.43)** | **<0.01** |
| ≥90 | 1.0 (ref) | **1.25 (1.04, 1.50)** | **1.31 (1.09, 1.59)** | **<0.01** |
| α-klotho |  |  |  |  |
| Age |  |  |  |  |
| < 54 | 1.0 (ref) | 1.02 (0.81, 1.30) | 0.90 (0.71, 1.13) | 0.36 |
| ≥54 | 1.0 (ref) | 0.87 (0.68, 1.10) | **0.79 (0.63, 1.00)** | **0.05** |
| Sex |  |  |  |  |
| Male | 1.0 (ref) | 0.92 (0.72, 1.18) | 0.95 (0.75, 1.21) | 0.69 |
| Female | 1.0 (ref) | 1.04 (0.83, 1.31) | **0.76 (0.61, 0.95)** | **0.02** |
| Smoking status |  |  |  |  |
| Yes | 1.0 (ref) | 0.83 (0.62, 1.12) | 0.84 (0.63, 1.13) | 0.25 |
| No | 1.0 (ref) | 1.12 (0.91, 1.39) | 0.86 (0.70, 1.06) | 0.17 |
| eGFR |  |  |  |  |
| < 90 | 1.0 (ref) | 1.16 (0.77, 1.74) | 0.95 (0.64, 1.43) | 0.85 |
| ≥90 | 1.0 (ref) | 0.95 (0.79, 1.14) | 0.87 (0.72, 1.04) | 0.12 |
| FGF23/α-klotho |  |  |  |  |
| Age |  |  |  |  |
| < 54 | 1.0 (ref) | 1.01 (0.80, 1.27) | 1.20 (0.96, 1.51) | 0.12 |
| ≥54 | 1.0 (ref) | **1.48 (1.18, 1.84)** | **1.63 (1.30, 2.03)** | **<0.01** |
| Sex |  |  |  |  |
| Male | 1.0 (ref) | **1.27 (1.00, 1.62)** | **1.47 (1.16, 1.87)** | **<0.01** |
| Female | 1.0 (ref) | **1.29 (1.03, 1.63)** | **1.32 (1.05, 1.67)** | **0.02** |
| Smoking status |  |  |  |  |
| Yes | 1.0 (ref) | 1.21 (0.90, 1.61) | **1.51 (1.13, 2.02)** | **<0.01** |
| No | 1.0 (ref) | **1.25 (1.02, 1.54)** | **1.30 (1.05, 1.60)** | **0.02** |
| eGFR |  |  |  |  |
| < 90 | 1.0 (ref) | 1.42 (0.96, 2.11) | **1.67 (1.13, 2.46)** | **0.01** |
| ≥90 | 1.0 (ref) | 1.18 (0.99, 1.42) | **1.29 (1.08, 1.56)** | **<0.01** |

Stratified analysis of odds ratios (95% confidence intervals [CIs]) of T2DM combined with CIMT according to tertiles of serum FGF23, α-klotho levels, and FGF23/α-klotho ratio.

Abbreviation: T2DM, type 2 diabetic mellitus; FGF23, fibroblast growth factor 23; α-klotho, α-membrane binding receptor Klotho. Models were adjusted for age, sex, smoking status, and BMI, SBP, DBP, TC, TG, HDL-c, LDL-c, and eGFR.

^a^ *P*-trend was obtained by including the median of each tertile (log_10_-transformed) of serum FGF23, α-klotho and FGF23/α-klotho ratio as a continuous variable in the logistic regression models.

Bold font indicates statistically significant.

|  | Tertiles | | | |
| --- | --- | --- | --- | --- |
|  | T1 | T2 | T3 | *P*-trend ^a^ |
| FGF23 |  |  |  |  |
| Age |  |  |  |  |
| < 54 | 1.0 (ref) | 1.10 (0.98, 1.24) | 1.11 (0.99, 1.24) | 0.09 |
| ≥54 | 1.0 (ref) | 1.04 (0.92, 1.18) | **1.15 (1.02, 1.31)** | **0.03** |
| Sex |  |  |  |  |
| Male | 1.0 (ref) | **1.17 (1.03, 1.32)** | **1.26 (1.11, 1.42)** | **<0.01** |
| Female | 1.0 (ref) | 1.01 (0.89, 1.14) | 1.08 (0.95, 1.21) | 0.23 |
| Smoking status |  |  |  |  |
| Yes | 1.0 (ref) | 1.12 (0.96, 1.31) | **1.17 (1.00, 1.37)** | **0.05** |
| No | 1.0 (ref) | 1.04 (0.93, 1.16) | **1.15 (1.03, 1.29)** | **0.01** |
| eGFR |  |  |  |  |
| < 90 | 1.0 (ref) | 1.19 (0.98, 1.46) | **1.51 (1.23, 1.86)** | **<0.01** |
| ≥90 | 1.0 (ref) | 1.06 (0.96, 1.16) | 1.09 (0.99, 1.20) | 0.08 |
| α-klotho |  |  |  |  |
| Age |  |  |  |  |
| < 54 | 1.0 (ref) | 1.04 (0.93, 1.17) | 0.98 (0.87, 1.10) | 0.68 |
| ≥54 | 1.0 (ref) | 0.95 (0.84, 1.08) | **0.88 (0.78, 0.99)** | **0.04** |
| Sex |  |  |  |  |
| Male | 1.0 (ref) | 1.02 (0.90, 1.15) | 1.02 (0.90, 1.16) | 0.74 |
| Female | 1.0 (ref) | 1.00 (0.88, 1.22) | **0.86 (0.76, 0.96)** | **0.01** |
| Smoking status |  |  |  |  |
| Yes | 1.0 (ref) | 0.98 (0.84, 1.13) | 0.97 (0.84, 1.13) | 0.72 |
| No | 1.0 (ref) | 1.05 (0.94, 1.17) | 0.91 (0.82, 1.02) | 0.10 |
| eGFR |  |  |  |  |
| < 90 | 1.0 (ref) | 1.09 (0.88, 1.36) | 1.03 (0.82, 1.28) | 0.79 |
| ≥90 | 1.0 (ref) | 1.00 (0.91, 1.10) | 0.94 (0.85, 1.03) | 0.18 |
| FGF23/α-klotho |  |  |  |  |
| Age |  |  |  |  |
| < 54 | 1.0 (ref) | 1.01 (0.90, 1.13) | 1.08 (0.97, 1.21) | 0.16 |
| ≥54 | 1.0 (ref) | **1.16 (1.03, 1.31)** | **1.21 (1.07, 1.36)** | **<0.01** |
| Sex |  |  |  |  |
| Male | 1.0 (ref) | 1.09 (0.96, 1.23) | **1.14 (1.01, 1.30)** | **0.04** |
| Female | 1.0 (ref) | 1.08 (0.96, 1.23) | **1.16 (1.03, 1.31)** | **0.02** |
| Smoking status |  |  |  |  |
| Yes | 1.0 (ref) | 1.06 (0.92, 1.24) | **1.16 (1.00, 1.34)** | **0.05** |
| No | 1.0 (ref) | 1.08 (0.97, 1.21) | **1.13 (1.01, 1.26)** | **0.03** |
| eGFR |  |  |  |  |
| < 90 | 1.0 (ref) | 1.06 (0.86, 1.32) | 1.23 (0.99, 1.53) | 0.06 |
| ≥90 | 1.0 (ref) | 1.07 (0.97, 1.17) | **1.10 (1.00, 1.21)** | **0.05** |

**Table S6** Stratified analysis of odds ratios (95% confidence intervals [CIs]) of T2DM combined with atherosclerosis according to tertiles of serum FGF23, α-klotho levels, and FGF23/α-klotho ratio.

Abbreviation: T2DM, type 2 diabetic mellitus; FGF23, fibroblast growth factor 23; α-klotho, α-membrane binding receptor Klotho. Models were adjusted for age, sex, smoking status, and BMI, SBP, DBP, TC, TG, HDL-c, LDL-c, and eGFR.

a P-trend was obtained by including the median of each tertile (log10-transformed) of serum FGF23, α-klotho and FGF23/α-klotho ratio as a continuous variable in the logistic regression models.

Bold font indicates statistically significant.
